# The nonsense-mediated mRNA decay factor Upf3 negatively regulates bulk autophagy progression in *Saccharomyces cerevisiae*

**DOI:** 10.1080/27694127.2026.2623730

**Published:** 2026-02-06

**Authors:** Tabassum Ahmad Tasmi, Emily Solomon, Emmanuella Wesome Avogo, Swaroopa Badenahalli Narasimhaiah, Elizabeth Delorme-Axford

**Affiliations:** Department of Biological Sciences, Oakland University, Rochester, MI, USA

**Keywords:** Atg16, autophagosome, macroautophagy, NMD, stress, yeast

## Abstract

Macroautophagy/Autophagy is a highly conserved mechanism that targets cytoplasmic cargo for degradation and recycling. At present, 45 autophagy-related (*ATG*) genes have been identified in fungi. Due to this complexity, the autophagy pathway must be strictly regulated at multiple levels (transcriptional, post-transcriptional, translational, and post-translational). Dysregulation of autophagy can have detrimental effects on cell health and survival. Therefore, investigation into the mechanisms regulating autophagy is critical. The nonsense-mediated mRNA decay (NMD) pathway targets transcripts with premature translation termination codons (PTCs), although NMD also regulates normal transcripts. NMD requires conserved factors in yeast – Upf1, Upf2, and Upf3. Here, we demonstrate that autophagy activity increases in *upf1*∆ *upf2*∆ *upf3*∆ cells. We also show that autophagy is enhanced in *upf3*∆ cells through multiple assays. *UPF3*/Upf3 expression decreases during starvation and autophagy induction. Loss of *UPF3* results in the upregulation of *ATG16*/Atg16, which is required for autophagosome formation. Furthermore, *ATG16* is likely targeted by NMD. These findings provide insight into how yeast cells may modulate autophagy through the mRNA decay factor Upf3.

## Introduction

Macroautophagy/Autophagy is a highly conserved process of cellular self-eating that targets cytoplasmic cargo for degradation and recycling. Autophagy occurs at a basal level to maintain cellular homeostasis but is markedly upregulated during stress conditions (such as nutrient deprivation or pathogen infection). Currently, 45 autophagy-related (*ATG*) genes have been identified in fungi, many of which have homologs or at least functional counterparts in more complex eukaryotes. Due to this inherent complexity, the autophagy pathway must be strictly modulated at multiple levels (transcriptional, post-transcriptional, translational, and post-translational). Dysregulation of autophagy (i.e., too much or too little) can have detrimental effects on cell health and survival. Notably, aberrant autophagy is associated with diverse human pathologies, such as cancer, neurodegenerative diseases, and lysosomal storage disorders. Due to the complexity underlying autophagy regulation, and because autophagy dysregulation contributes to disease,^[1]^ it is crucial to understand how these genes are modulated. Therefore, investigation into the precise molecular mechanisms regulating *ATG* genes is of utmost importance.

Cellular RNA decay pathways regulate basal gene expression and RNA quality control (reviewed in Delorme-Axford and Klionsky)^[2]^. Our previous work demonstrated that select *ATGs* are targeted by Xrn1, the major 5‘−3‘ cytoplasmic exoribonuclease, under nutrient-rich conditions in yeast^[3]^. However, it is unknown why select *ATGs* (and not others) are regulated by Xrn1. Interestingly, Xrn1 functions as a central downstream ribonuclease in the nonsense-mediated mRNA decay (NMD) pathway^[4,5]^. Nonsense-mediated decay is an evolutionarily conserved mechanism for RNA degradation,^[6]^ serving both quality control and regulatory functions. NMD maintains quality control to eliminate transcripts with premature termination codons (PTCs)^[7]^. In fact, PTCs introduced into transcripts by nonsense or frameshift mutations cause one-third of inherited human diseases^[8]^. However, NMD also targets a significant proportion of physiologically normal WT mRNA substrates^[9]^. Consequently, NMD serves as a regulatory mechanism for the selection and degradation of transcripts within the cell, thereby modulating gene expression. Structural features that may confer substrate selection include the presence of long 3’ untranslated regions (UTRs), short 5’ UTR reading frames, and upstream open reading frames (uORFs)^[6,10–12]^. Additional recent evidence also indicates a role for the cellular environment^[13,14]^. However, the precise molecular mechanisms governing this targeting are still not fully understood.

Transcript targeting by NMD requires three conserved factors, Nam7/UPF1 (yeast/human), Nmd2/UPF2, and Upf3/UPF3B^[15–18]^. Single or multiple deletions of either *UPF1*, *UPF2*, or *UPF3* in yeast inhibit NMD to a similar extent^[15,19,20]^. Although the mammalian system has two UPF3 homologs, evidence indicates that the paralogs UPF3A/UPF3 and UPF3B/UPF3X may have antagonistic^[21]^ and/or redundant functions^[22]^. UPF3B exhibits strong NMD activity^[21,23]^, whereas UPF3A only weakly activates NMD in the presence of UPF3B^[21,22]^. While all three Upf factors (Upf1, Upf2, and Upf3) are required for NMD in yeast, UPF3B may be dispensable for NMD in mammals^[22]^. In the absence of UPF3B, UPF3A may compensate for NMD^[22,24]^. In the absence of both UPF3A and UPF3B, mRNAs are still targeted to NMD but with lower efficiency^[22]^.

Both human UPF3A and UPF3B contain the N-terminal putative ribonucleoprotein domain (also known as an RNA recognition motif/RRM^[25,26]^). For both UPF3A and UPF3B, the central RNA recognition motif binds to UPF2 (reviewed in He and Jacobson^[11]^). However, only recently have mRNA and ribosome binding to UPF3B been reported^[27,28]^. Furthermore, NMD is implicated in the pathogenesis of human diseases, including cancer and other genetic diseases^[29–32]^. In fact, mutations in the human *UPF3* homologs were identified in individuals with neurodevelopmental disorders^[33–35]^ and autism (reviewed in Yi et al.^[10]^).

Previous work by Wengrod and colleagues demonstrated that depletion of UPF1 or UPF2 enhances autophagy in mammalian U2OS osteosarcoma cells^[36]^. However, the role(s) of UPF3A or UPF3B were not investigated in the aforementioned study^[36]^. Moreover, no homologous pathway linking autophagy and NMD in yeast has been identified^[2]^, and to the best of our knowledge, no one has examined the role of Upf3 in autophagy in yeast. To address the gap in our understanding of whether Upf3 functions in autophagy^[37]^ and to expand on prior work^[3,36]^, we investigated the role of Upf3 in autophagy.

In this study, we find that simultaneous deletion of all three yeast NMD factors (*UPF1*, *UPF2*, and *UPF3*) enhances Atg8 lipidation and GFP-Atg8 processing. Furthermore, chromosomal deletion of *UPF3* alone enhances autophagy through multiple assays. *UPF3*/Upf3 expression decreases when cells are starved for nitrogen (a robust activator of autophagy activity). Additionally, loss of *UPF3* results in the upregulation of *ATG16*/Atg16, which is required for autophagosome formation. These findings provide insight into how yeast cells may modulate autophagy through the NMD factor Upf3.

## Results

### Autophagy activity increases in the absence of nonsense-mediated mRNA decay factors in *Saccharomyces cerevisiae*

**Figure 1.**
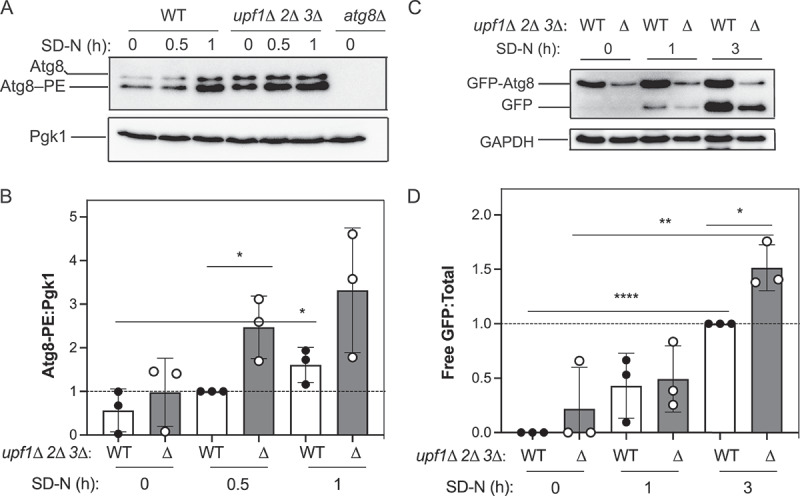
Loss of NMD factors enhances autophagy in yeast. (A) Monitoring the total amount of Atg8 and its lipidation status is a readout for autophagy induction. WT (WLY176) and *upf1*Δ *upf2*Δ *upf3*Δ (EDA283), and *atg8*Δ (YAB369) strains were assayed for Atg8 lipidation (Atg8–PE) at 0, 0.5, and 1 h of starvation (SD-N). Protein extracts were analyzed by urea SDS-PAGE and blotted with anti-Atg8 or anti-Pgk1 (loading control) antibodies. The blot shown is representative of three independent experiments. (B) Densitometry of blots represented in (A). The ratio of lipidated Atg8 (Atg8–PE):Pgk1 was quantified (*n* = 3). (C) WT (EDA284) and *upf1*Δ *upf2*Δ *upf3*Δ (EDA285) strains expressing a chromosomally integrated plasmid encoding GFP-Atg8 were grown to mid-log phase in rich media and then starved for nitrogen (SD-N medium) for 0, 1, and 3 h. Protein extracts were analyzed by SDS-PAGE and blotted with anti-GFP or anti-GAPDH (loading control) antibodies. A representative blot is shown (*n* = 3). (D) Processed GFP-Atg8 was calculated by determining the ratio of free GFP:total GFP-Atg8 (sum of free GFP and full-length GFP-Atg8). Results shown are relative to the level of the WT strain during starvation (3 h SD-N), which was set to 1 (*n* = 3). In (B) and (D), error bars represent standard deviation (SD, *p < 0.05; **p < 0.01; ****p < 0.0001). Also see Tables S1 and S2.

Components of the RNA decay machinery have been identified as post-transcriptional autophagy regulators^[3,38–40]^. Following up on this prior work, we examined whether RNA decay factors from the NMD pathway – Upf1, Upf2, and Upf3 – regulate autophagy activity in yeast. To investigate whether NMD factors may modulate autophagy, we examined a yeast strain lacking *UPF1*, *UPF2*, and *UPF3* (Figure 1A,B) for Atg8 lipidation. Atg8 is required for autophagy^[41]^ and associates with the inner and outer membranes of the initial sequestering compartment, the phagophore; the Atg8 on the outer surface of the completed phagophore – the autophagosome – is removed prior to fusion with the vacuole^[42]^. In the cell, Atg8 exists as two species – a non-lipidated soluble species and a lipidated phosphatidylethanolamine (PE)-conjugated membrane-associated species^[43]^. In addition, increased *ATG8* mRNA and Atg8 protein levels correlate with higher autophagy activity^[44]^. Furthermore, nitrogen starvation is a robust activator of autophagy in yeast^[45]^. As expected, increased Atg8 lipidation (Atg8–PE) was observed in the wild-type (WT) strain with increasing time points of nitrogen starvation (Figure 1A,B). A complete absence of bands was noted in the negative control *atg8∆* strain, indicating that the observed bands were bona fide Atg8. In the *upf1∆ upf2∆ upf3∆* strain, we observed an increased amount of Atg8 lipidation (Atg8–PE) in starvation conditions relative to the WT strain (Figure 1A,B), supporting the idea that the loss of NMD factors enhances autophagy.

As an additional method, we also assessed autophagy flux in *upf1∆ upf2∆ upf3∆* cells using the GFP-Atg8 processing assay (Figure 1C,D). The GFP-Atg8 assay is a quantitative method to monitor autophagy progression^[43]^. To monitor the delivery of Atg8 to the vacuole, Atg8 is N-terminally tagged with GFP; C-terminal tagging will be lost due to the action of the protease Atg4 on Atg8^[46]^. The vacuolar delivery of GFP-Atg8 is monitored with western blotting. Bulk autophagy results in an accumulation of free GFP in the vacuole because GFP is more resistant to vacuolar degradation than Atg8. Thus, the GFP-Atg8 assay is a method to assess nonselective autophagy based on the release of free GFP; increasing amounts of free GFP correspond to a greater degree of autophagy flux^[45,47]^. Here, we used cells expressing GFP-Atg8 under control of the *ATG8* promoter (Figure 1C,D). As expected, we observed enhanced GFP-Atg8 processing as indicated by the accumulation of free GFP when *upf1∆ upf2∆ upf3∆* cells were starved for nitrogen for 3 h, compared to WT (~1.5-fold; Figure 1C,D). Given that Atg8 lipidation and GFP-Atg8 processing were enhanced in the *upf1*Δ *upf2*Δ *upf3*Δ strain and others have investigated UPF1 and UPF2 in mammalian cells^[36]^, we focused on the role of Upf3 in autophagy in yeast.

### Loss of the nonsense-mediated mRNA decay factor UPF3 enhances autophagy

Analysis of the amino acid sequence of yeast Upf3 establishes important functional features (Figure S1A). Upf3 has three nuclear localization signals (NLSs) at Lys15 to Lys31, Arg58 to Ala75, and Lys284 to Arg300^[26,48]^; although one study identified the second NLS within residues Arg58 to Lys74^[26]^, rather than Ala75^[48]^. Nuclear export signals/NESs reside within residues Leu88–Leu97 and Leu151–Lys160^[20]^. The Upf3 interaction domain with Upf2 has been mapped within residues Gly78 to Lys278^[15]^.

**Figure 2.**
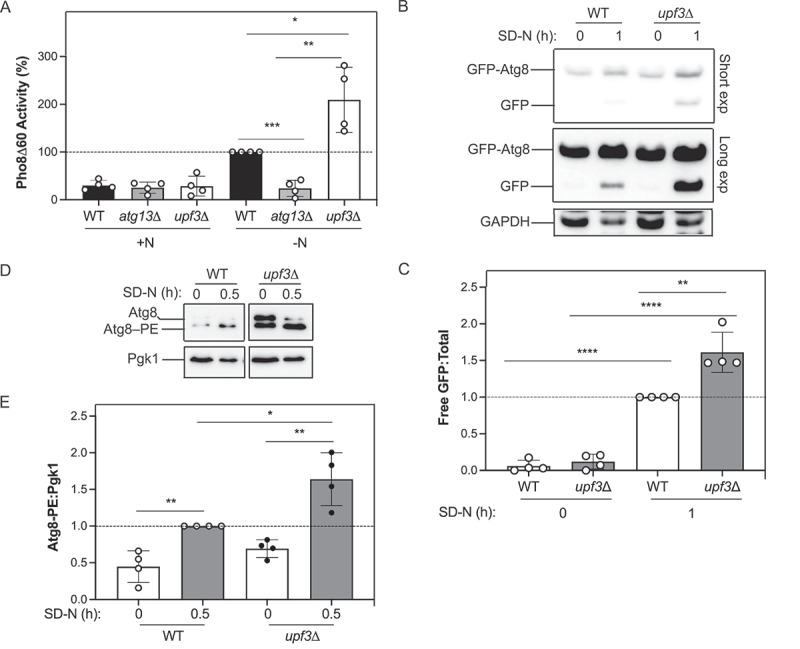
Loss of *UPF3* enhances nonselective autophagy. (A) WT (JMY347), *atg13*Δ (EDA328), and *upf3*Δ (EDA302) cells were grown to mid-log phase in YPD (+N) and then starved for nitrogen (–N) for 2.5 h. The Pho8Δ60 activity was measured and normalized to the activity of starved WT cells at 2.5 h, which was set at 100% (*n* = 4). (B) WT (JMY347) and *upf3*Δ (EDA302) cells expressing GFP-Atg8 were grown to mid-log phase in rich media and then starved for nitrogen (SD-N medium) for 0 and 1 h. Protein extracts were analyzed by SDS-PAGE and blotted with anti-GFP or anti-GAPDH (loading control) antibodies (*n* = 4). Two exposures are included for GFP (“short” and “long”). (C) Processed GFP-Atg8 was calculated by determining the ratio of GFP:total GFP-Atg8 (sum of free GFP and full-length GFP-Atg8). Results shown are relative to the WT strain during starvation (1 h SD-N), which was set to 1 (*n* = 4). (D) WT (WLY176) and *upf3*Δ (EDA164) strains were assayed for Atg8 at 0 and 0.5 h of starvation (SD-N). Protein extracts were analyzed by urea SDS-PAGE and blotted with anti-Atg8 or anti-Pgk1 (loading control) antibodies. The blot was cropped to show the relevant lanes. A representative blot is shown (*n* = 4). (E) Densitometry of blots represented in (D). The ratio of lipidated Atg8 (Atg8–PE):Pgk1 was quantified (*n* = 4). For (A), (C), and (E), error bars represent SD (*p < 0.05; **p < 0.01; ***p < 0.001; ****p < 0.0001). Also see Tables S1 and S2.

To assess autophagy flux, we performed the modified vacuolar alkaline phosphatase, or Pho8Δ60 assay (Figure 2A), a quantitative enzymatic method to measure autophagy flux by assessing vacuolar phosphatase activity^[49]^. Pho8 is a resident vacuolar hydrolase; Pho8Δ60 is an N-terminal truncated version of Pho8 wherein the phosphatase is only trafficked to the vacuole during autophagy^[49]^. When nonselective autophagy is induced, a portion of the cytosol is engulfed by the phagophore, and then delivered to the vacuole for degradation following the fusion of the latter with the autophagosome. As a result, Pho8Δ60 is also sequestered within the autophagosome and transported by the vacuole where it is processed into its enzymatically active form^[47]^. Pho8Δ60-dependent phosphatase activity will thus be directly proportional to the amount of cytosol delivered to the vacuole^[49]^. Some minimal degree of basal phosphatase activity is observed in cells under nutrient-rich conditions^[3]^. When cells were starved for nitrogen (2.5 h), WT cells showed enhanced autophagy activity as expected (Figure 2A). We chose 2.5 h because it is the shortest timepoint we have tested that produces a robust level of Pho8Δ60 activity with reduced background activity^[50]^. Little autophagy activity was seen in the negative control *atg13*Δ strain (~24%). Atg13 is a component of the initial protein complex recruited to the phagophore assembly site (PAS), the site of autophagosome formation, is important for autophagy initiation (reviewed in Refs.^[45,51]^) and is essential for autophagy in yeast^[52]^. Autophagy was enhanced at ~2-fold higher levels in the *upf3*Δ strain compared to the WT during nitrogen starvation (Figure 2A).

We also assessed autophagy flux in *upf3*Δ cells using the GFP-Atg8 processing assay (Figures 2B,C and S1B,C). Here, we used cells expressing GFP-Atg8 under control of the copper promoter to eliminate any potential Upf3-dependent effects on Atg8 expression (Figure 2B,C). We observed enhanced GFP-Atg8 processing as indicated by the accumulation of free GFP when *upf3*Δ cells were starved for nitrogen for 1 h, compared to WT (~2-fold; Figure 2B,C). As an additional approach, we also evaluated cells expressing GFP-Atg8 under control of the *ATG8* promoter (Figure S1B,C). We noted increased GFP-Atg8 processing and higher levels of free GFP in starved *upf3*∆ cells compared to the WT strain (Figure S1B,C). Furthermore, we also examined the Atg8 lipidation status of *upf3*Δ cells (Figure 2D,E). When cells lacking *UPF3* were starved for nitrogen, we observed a greater ratio of lipidated Atg8–PE compared to WT cells (Figure 2D,E). Taken together, our data support the idea that loss of *UPF3* enhances autophagy in yeast.

### Upf3 expression decreases with nitrogen starvation and autophagy induction

**Figure 3.**
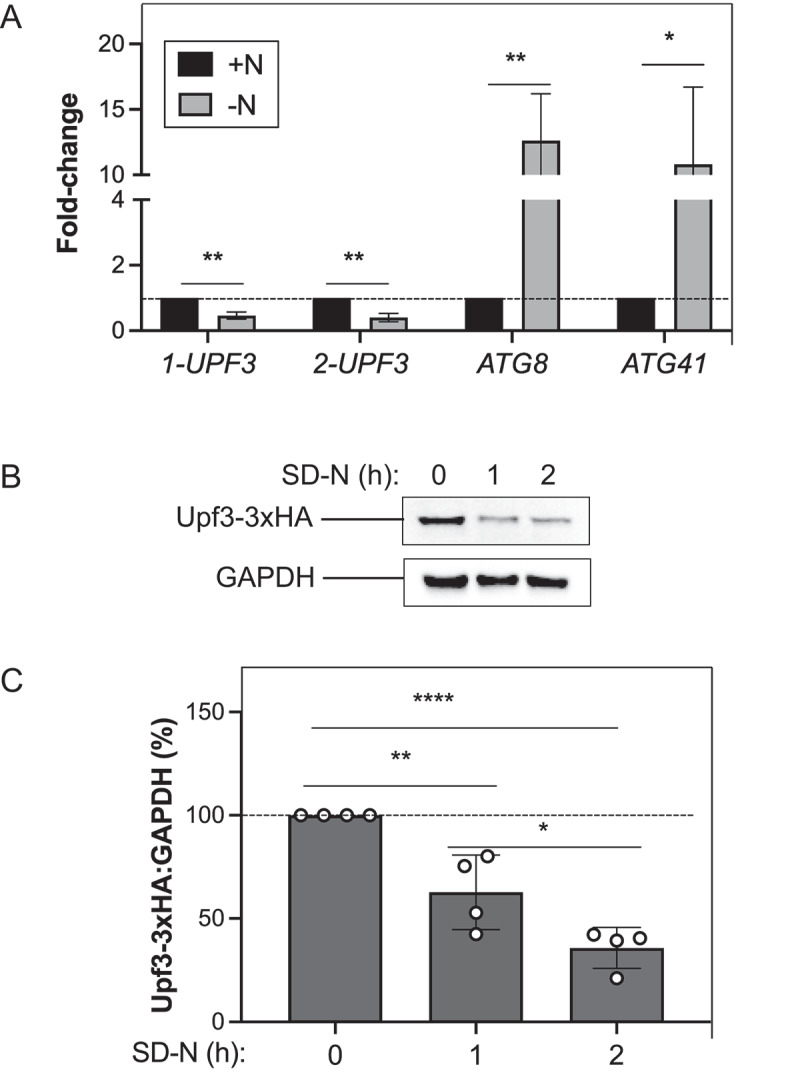
Upf3 expression levels decrease after autophagy induction. (A) WT (SEY6210) cells were grown to mid-log phase in YPD (+N) and then nitrogen starved (–N) for 1 h. Total RNA was extracted, and RT-qPCR was performed. Results shown are relative to the level of WT in rich conditions (+N), which was set to 1. The geometric mean of *TFC1* and *SLD3* was used to quantify relative expression levels (*n* = 3). (B) Upf3-3xHA fusion protein levels decrease after autophagy induction. Cells endogenously expressing Upf3-3xHA (EWA013) were grown in YPD to mid-log phase and then starved (SD-N) for 0, 1, or 2 h. Protein extracts were analyzed by SDS-PAGE and blotted with anti-HA or anti-GAPDH (loading control) antibodies. A representative blot is shown (*n* = 4). (C) Densitometry of blots represented in (B). The percentage of Upf3-3xHA:GAPDH was quantified (*n* = 4). For (A) and (C), error bars represent SD (*p < 0.05; **p < 0.01; **** *p* < 0.0001). Also see Tables S1, S2, and S3.

Factors that negatively regulate autophagy under nutrient-rich conditions are typically inactivated following autophagy induction^[53]^. We first examined *UPF3* mRNA levels during both nutrient-rich and nitrogen-starvation conditions in WT cells with real-time quantitative PCR (RT-qPCR; Figure 3A). As positive controls, we also assessed *ATG8* and *ATG41* expression. *ATG8*/Atg8 and *ATG41*/Atg41 mRNA and protein levels increase during autophagy^[44,54]^; Atg41 is required for autophagy and mitophagy^[54,55]^. As expected, we observed a significant upregulation of *ATG8* and *ATG41* during nitrogen starvation compared to nutrient-rich conditions (Figure 3A). In contrast, we observed a significant decrease (>50%) in *UPF3* mRNA levels at 1 h of nitrogen starvation (Figure 3A).

To determine whether the expression of Upf3 protein was altered during nitrogen starvation and autophagy, we chromosomally tagged *UPF3* at its C terminus with 3xHA (Figure 3B,C) or protein A (PA; Figure S2) and assessed the fusion protein expression levels by western blot analysis. We observed a significant decrease in Upf3-3xHA fusion protein expression during the time course of nitrogen starvation (~40% by 1 h and >60% by 2 h; Figure 3B,C). Upf3-PA fusion protein expression also decreased significantly during the same time course of nitrogen starvation (Figure S2). Taken together, these data support that *UPF3* mRNA and Upf3 fusion protein levels decrease during nitrogen starvation when autophagy is stimulated.

### Upf3 negatively regulates *ATG16* expression

**Figure 4.**
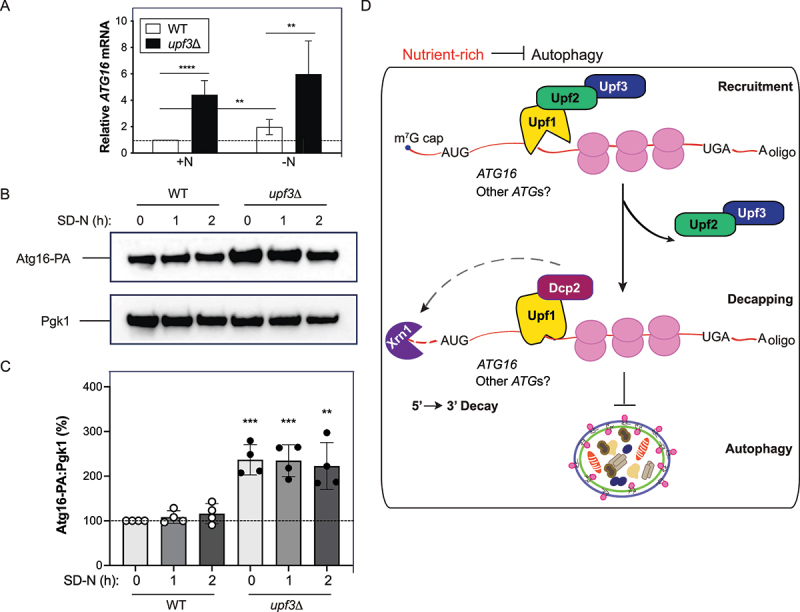
Upf3 negatively regulates *ATG16*/Atg16. (A) Upf3 negatively regulates the expression of *ATG16*. WT (WLY176) and *upf3*Δ (EDA164) cells were grown to mid-log phase in YPD (+N). Total RNA was extracted, and RT-qPCR was performed. Results are shown relative to the level of *ATG16* mRNA expression in WT cells under rich conditions (+N), which was set to 1. The geometric mean of *TFC1* and *SLD3* were used to quantify relative expression levels. Results shown are the mean of five independent experiments. (B) Loss of *UPF3* enhances Atg16-PA fusion protein expression. WT (EDA325) and *upf3*Δ (EDA327) cells endogenously expressing Atg16-PA were grown to mid-log phase in YPD and then nitrogen-starved (SD-N) for 0, 1, or 2 h. Protein extracts were analyzed by SDS-PAGE and blotted with anti-PA or anti-Pgk1 (loading control) antibodies (*n* = 4). (C) Densitometry of blots represented in (B). The percentage of Atg16-PA:Pgk1 was quantified (*n* = 4). For (A) and (C), error bars represent SD (**p < 0.01; ***p < 0.001; **** *p* < 0.0001). (D) Schematic representation of the model by which Upf3 functions as a negative regulator of bulk autophagy progression in *S. cerevisiae*. In WT cells under nutrient-rich conditions, *ATG16*, and potentially other *ATG*s^[57]^, undergo nonsense-mediated mRNA decay, and autophagy is maintained at a basal level. Note that not all factors involved in RNA degradation are shown. Also see Tables S1, S2, and S3.

As Upf3 is a fundamental factor for NMD^[19,56]^ and autophagy is enhanced in *upf3∆* cells (Figure 2 and Figure S1B,C), we examined whether loss of *UPF3* affected *ATG* mRNA levels by RT-qPCR (Figure S3A,B). Under nutrient-rich conditions, we observed that the mRNA levels of several of the *ATG* genes we examined displayed a general trend of increasing in the *upf3∆* strain compared to WT, although the differences were not statistically significant (Figure S3A,B). The one exception was seen with *ATG16*, which was significantly upregulated in *upf3*Δ cells (>4-fold; Figures 4A and S3C). Under nitrogen-starved conditions, *ATG16* levels remained elevated in *upf3*Δ cells (~6-fold) compared to WT cells (Figure 4A). Deletion of yeast NMD factors *UPF1*, *UPF2*, or *UPF3* generally leads to the stabilization of the same repertoire of mRNA targets^[15,57,58]^. Accordingly, we also examined whether *ATG16* mRNA levels were enhanced in *upf1*Δ or *upf2*Δ cells (Figure S3C). During both nutrient-rich and nitrogen starved conditions, we found that *ATG16* mRNA levels were also upregulated in cells lacking *UPF1* or *UPF2* (Figure S3C). RNA-seq profiling by Celik et al. showed that *ATG16* is upregulated in the absence of *UPF1*, *UPF2*, or *UPF3*^[57]^, consistent with our results (Figure S3C).

The coiled-coil protein Atg16 is essential for autophagy^[59]^. As *ATG16* mRNA expression was enhanced in the absence of *UPF3*, we examined whether Atg16 exhibited a corresponding increase at the protein level (Figures 4B,C and S3D). We chromosomally tagged *ATG16* at its C terminus with PA and assessed the fusion protein expression levels by western blot (Figures 4B,C and S3D). In *upf3*Δ cells, we observed a significant increase in Atg16-PA fusion protein levels under nutrient-rich and starved conditions compared to WT (Figures 4B,C and S3D), consistent with our RT-qPCR results (Figure 4A). At 1 and 2 h of nitrogen starvation, the Atg16-PA fusion protein levels remained elevated relative to those in the WT (> 200%; Figures 4B,C and S3D). These results support a role for Upf3 as a negative regulator of *ATG16*/Atg16 expression.

Previous work in yeast demonstrated that Atg8 levels directly control autophagosome size, thereby increasing autophagy^[44]^. However, to the best of our knowledge, we did not identify any other studies in yeast that demonstrated a relationship between Atg16 levels and autophagy activity (similar to what has been shown for Atg8^[44]^). Given that the loss of *UPF3* increases both autophagy activity (Figures 2 and S1B,C) and *ATG16*/Atg16 levels (Figures 4A–C and S3C,D), we further examined whether Atg16 overexpression in yeast was sufficient to enhance autophagy activity. To further investigate the impact of upregulated Atg16 levels on autophagy, *ATG16* was overexpressed under the control of the *ZEO1* promoter (Figure S4A). As expected, *atg1*Δ cells (Atg1 is a serine/threonine kinase required for autophagy^[60]^) showed significantly lower Pho8Δ60 activity compared to WT cells expressing Atg16-PA (Figure S4B). This finding also demonstrates that the Atg16-PA fusion protein is still functional in autophagy, consistent with the findings of others using C-terminal tagged Atg16 fusion proteins^[59]^. Overexpression of Atg16 under the *ZEO1* promoter slightly reduced Pho8Δ60 activity (>15%) compared to WT cells endogenously expressing Atg16-PA (Figure S4B).

Similar to our results, prior work in cell lines has shown that the overexpression of ATG16L1 inhibits autophagy, likely due to stoichiometric alterations^[61,62]^. To note, the overexpression of *ATG16* under the control of the *ZEO1* promoter resulted in a considerable increase in Atg16-PA fusion protein expression (Figure S4A); this contrasts with the comparatively milder enhancement of *ATG16*/Atg16 observed in the absence of *UPF3* (Figures 4A–C and S3C,D). Therefore, it is plausible that a moderate upregulation in Atg16 could enhance autophagy (such as in the absence of *UPF3*), whereas a greater increase in Atg16 protein levels (such as under the control of the *ZEO1* promoter) is detrimental due to stoichiometric disruptions as proposed by others^[61,62]^. This is consistent with the idea that the magnitude of the autophagic response is likely affected by the stoichiometric ratio of Atg proteins^[3]^.

## Discussion

Here, we present data supporting a model for the NMD factor Upf3 as a regulator of autophagy and *ATG16*/Atg16 expression in yeast (Figure 4D). Chromosomal deletion of *UPF3* resulted in a significant upregulation of autophagy when assessed by multiple assays. We also found that *UPF3* mRNA and Upf3 protein levels were significantly decreased during nitrogen starvation and autophagy induction. Our data further indicate that both *ATG16* mRNA and Atg16 fusion protein levels are higher in *upf3*Δ cells. During starvation conditions, *ATG16* mRNA and Atg16 fusion protein levels remain elevated in the absence of *UPF3*, further supporting that Upf3 functions as a negative regulator of *ATG16*/Atg16 expression.

Atg16 is a subunit of the Atg12–Atg5–Atg16 complex required for Atg8 lipidation^[63]^. Atg16 interacts directly with Atg5 via its N-terminal Atg5-interacting motif^[59,64]^. Atg16 binds to membranes via its amphipathic α-helix (residues 22–46)^[65]^ and also binds to Atg21^[66,67]^. Atg21 is a phosphoinositide binding protein that is required for autophagy and organizes the Atg12–Atg5–Atg16 complex and Atg8 at the PAS^[66]^. Based on our data presented above, loss of *UPF3* increases Atg16 levels, which is accompanied by an increase in autophagy activity.

As noted above, profiling analysis by Celik et al. showed that *ATG16* is upregulated in the absence of *UPF3* (~5-fold)^[57]^, consistent with our results. Also consistent with our results, no alterations in *ATG1*, *ATG7*, *ATG8*, *ATG9, ATG11*, *ATG14*, *ATG17*, or *ATG41/ICY2* were noted^[57]^. Celik and colleagues also demonstrated that certain *ATG* genes (which we did not examine in our study) were also enhanced (~2-fold above WT) in the absence of *UPF3*^[57]^. Therefore, it is possible that there may be other *ATG* transcripts affected in *upf3*Δ cells, beyond those that we have examined here, that could account for the upregulation of autophagy activity that is observed in the absence of *UPF3* (Figure 2).

Depending on the organism and cell type, approximately 5–20% of all transcripts are NMD substrates (reviewed in Ref.^[57]^). Transcripts targeted by NMD are identified by those whose abundance increases significantly upon NMD pathway inactivation (i.e., in *upf1*Δ, *upf2*Δ, and *upf3*Δ cells)^[68]^. Here, we show that *ATG16* expression is enhanced in *upf1*Δ, *upf2*Δ, and *upf3*Δ cells, consistent with others^[57]^, suggesting that *ATG16* is a target of the NMD pathway in yeast. Furthermore, the publicly available genomics platform Ensembl (www.ensembl.org)^[69]^ annotates the homolog – *ATG16L1* – as a target of NMD, although further experimental validation is needed.

In yeast, mRNAs targeted by NMD are primarily degraded through a deadenylation-independent mechanism involving decapping by Dcp2 (the decapping enzyme)^[57]^. NMD-regulated transcripts undergo decapping and degradation by 5’–3’ exonucleolytic decay mechanisms and demonstrate upregulated expression in cells lacking either *UPF1*, *UPF2*, *UPF3*, *XRN1*, or *DCP2*^[57]^. Prior work by Hu and colleagues showed that a strain harboring a temperature-sensitive (*ts*) mutation of *DCP2* (*dcp2-7*Δ) significant enhances *ATG16* levels, supporting that *ATG16* is regulated in a Dcp2-dependent manner^[38]^. Additionally, we previously noted that *ATG16* expression was upregulated in *xrn1*Δ cells under nutrient-rich conditions^[3]^. Xrn1 is the major cytoplasmic exonuclease for 5’–3’ RNA decay in yeast (reviewed in Ref.^[2]^); the majority of NMD substrates are targeted by Xrn1 in the 5’–3’ RNA decay pathway^[4]^. Given that *ATG16* expression is enhanced in *upf3*Δ (Figures 4A and S3C and Ref.^[57]^), *dcp2-7*Δ *ts*^[38]^, and *xrn1*Δ^[3]^ cells, *ATG16* is likely targeted by NMD, decapped by Dcp2, and then degraded by Xrn1 in the 5’–3’ exonucleolytic decay pathway.

Our model also aligns with a recent NMD study by Ruiz-Gutierrez and colleagues^[70]^. The RNA helicase Upf1 binds to NMD substrates^[71,72]^, recruiting the Upf2/Upf3 heterodimer^[70,73]^. Upf2 bridges the interaction between Upf1 and Upf3^[74]^. Dcp2 competes with Upf2 for the same binding site on Upf1^[70]^. An uncharacterized molecular switch displaces Upf2/Upf3 from Upf1, facilitating Dcp2 binding and assembly of the decapping complex^[70,73]^. Recruitment of the decapping machinery enables RNA degradation of NMD targets (including *ATG16* and potentially other *ATG*s^[57]^) to proceed, maintaining autophagy at basal levels in WT cells under nutrient-rich conditions (Figure 4D).

NMD is a highly conserved RNA degradation mechanism first described as a surveillance pathway for degrading mRNA transcripts with PTCs. NMD also regulates normal transcripts, although the mechanisms by which this selection occurs are not fully elucidated. RNA targets harboring long 3’ UTRs, short reading frames in 5’ UTRs, and uORFs may confer target selectivity, thereby targeting an otherwise physiologically normal substrate for degradation via NMD^[6,10–12]^. In addition, Celik et al. found that the majority of the NMD regulated transcripts derived from protein coding genes appeared to be “normal” mRNAs that lacked any readily discernible structural features that may have otherwise indicated they were NMD substrates^[57]^.

Kebaara et al. analyzed WT mRNAs in *S. cerevisiae* with longer than expected 3’ UTRs – 91% were degraded by NMD^[75]^. However, *ATG16* was not characterized among the mRNAs with long 3’ UTRs^[75]^. A recent study by Yang and colleagues identified uORFs in certain *ATG* genes that mediate protein translation^[76]^; *ATG16* was not identified to have a predicted canonical uORF. An additional structural feature that may confer susceptibility to NMD is the presence of a stop codon encountered within the +1 or +2 reading frame^[57]^. Further analysis into the coding sequence of *ATG16* reveals that the introduction of either a +1 or +2 frameshift results in the presence of multiple termination codons within either of these reading frames. Future work may be aimed at validating whether these features of *ATG16* confer its susceptibility to regulation by NMD. It is also possible that *ATG16* harbors other, as yet unidentified, features that make it a target for Upf3 and/or NMD targeting.

Alterations in the expression of autophagy machinery components, such as *ATG7*^[77]^, *ATG8*^[44]^, *ATG9*^[78]^, and *ATG41*^[54]^, have a direct impact on the magnitude of the autophagy response. Our work indicates that autophagy is enhanced in the absence of *UPF3*; this is accompanied by a corresponding increase in *ATG16*/Atg16 levels. However, we cannot rule out that other *ATG* transcripts may be enhanced in *upf3*Δ cells that could account for the corresponding increase in autophagy that we observed. In addition, it is possible that upregulation of *ATG16* in combination with multiple other *ATG* genes (such as those identified by Celik et al.^[57]^) may result in the enhanced autophagy response that we observed in *upf3*Δ cells. A similar combinatorial input from multiple *ATG* genes has been proposed for the phenotype observed in cells lacking the histone demethylase, Rph1^[79]^. Additionally, it is also possible that the bolstered autophagy that we noted in *upf3*Δ cells could be the result of NMD-mediated targeting of an as yet unidentified central upstream regulator. To note, in the study by Celik et al., neither target of rapamycin (TOR), the central regulator of autophagy^[80]^, or the transcriptional activator Gcn4, which regulates multiple *ATG* genes^[81]^, were altered to an appreciable degree in either *upf1*Δ, *upf2*Δ, or *upf3*Δ cells^[57]^.

We initially predicted that the moderate enhancement of *ATG16*/Atg16 levels in *upf3*Δ cells was driving the corresponding upregulation in autophagy, similar to other *ATG* genes^[44,54,77,78]^. However, we observed that substantial overexpression of *ATG16* (driven by the *ZEO1* promoter) resulted in a slight decrease in autophagy as assessed by Pho8Δ60 activity. This is consistent with the findings of others – that overexpression of ATG16L1 inhibits autophagy, likely due to stoichiometric alterations in the autophagy machinery^[61,62]^. Nevertheless, the upregulation of *ATG16*/Atg16 in *upf3*Δ cells was modest compared to the expression of *ATG16* under the *ZEO1* promoter. Consistent with the idea that the extent of the autophagic response is likely affected by the appropriate stoichiometric ratio of Atg proteins^[3]^, a mild augmentation in Atg16 protein levels (such as what we observe in *upf3*Δ cells) may have a positive impact on autophagy, whereas a massive upregulation in Atg16 is detrimental due to the greater degree of stoichiometric disruption.

Prior work by Wengrod and colleagues demonstrated that autophagy was increased in a mammalian cell line following depletion of UPF1 or UPF2 by short hairpin RNAs under basal, amino acid starvation, and rapamycin-treated conditions^[36]^. Wengrod et al. also observed that cell viability is decreased when both autophagy and NMD were inhibited^[36]^, indicating the importance of autophagy for cell survival when RNA quality control mechanisms are dysfunctional. However, prior to our study, no homologous mechanisms involving NMD factors had been previously identified as having an effect on autophagy in yeast.

Elucidating the regulatory mechanisms modulating autophagy and *ATG* gene expression continues to be an important area of study in the field, given its critical role in cell physiology^[82]^. Prior to our current work, there has been no prior known association between Upf3 (or its homologs) and autophagy in any model system. NMD is a quality-control mechanism that targets aberrant transcripts for decay, but also regulates otherwise normal transcripts. Here, we show that autophagy and *ATG16*/Atg16 expression is enhanced in *upf3*Δ cells, although the mechanism by which Upf3 regulates autophagy is not fully understood. Furthermore, *ATG16* is likely a substrate for NMD. Through the targeting of *ATG16* and potentially other *ATG* transcripts, NMD may also play a role in maintaining autophagy at the levels that are necessary for cellular needs. Further investigation into these mechanisms will enable us to better understand the complex interplay between autophagy and cellular RNA decay pathways.

## Materials and Methods

### Yeast strains, media, and cell culture

Yeast strains used in this study are listed in Table S1. Yeast cells were grown in YPD (1% yeast extract, 2% peptone, and 2% glucose) medium from BD Difco (BD 242820) or Gibco (A1374501). Autophagy was induced by shifting mid-log phase cells from rich medium to nitrogen-starvation medium (SD-N; 0.17% yeast nitrogen base without ammonium sulfate or amino acids and 2% glucose) for the indicated times. Gene deletions and chromosome tagging were performed using standard methods^[83,84]^.

### SDS-PAGE and western blotting

Atg8, GFP-Atg8, and other western blot assays were performed as previously described^[43,85]^. Briefly, 1 OD_600_ equivalent units were collected for western blot analysis. Proteins were precipitated using ice-cold 10% trichloroacetic acid, then washed with ice-cold acetone, dried, and stored at −20°C prior to analysis. Precipitated cell pellets were lysed by vortexing with acid-washed glass beads (Sigma, G8772) in MURB buffer (50 mM sodium phosphate, pH 7.0; 25 mM MES; 1% SDS [w:v]; 3 M urea; 1 mM NaN_3_; 1% β-mercaptoethanol; 0.01% bromophenol blue) for 5 min at 4°C. Samples were incubated at 55°C for 15 min, then centrifuged at 10,000 × *g* for 1 min. The supernatant was loaded on the appropriate SDS-PAGE gel (10–12.5% polyacrylamide). SDS-PAGE for Atg8 lipidation assays were performed using polyacrylamide gels containing urea to separate Atg8–PE from unconjugated Atg8^[43]^. SDS-PAGE was followed by a semi-dry transfer using a TransBlot SD Semi-Dry Transfer Cell (Bio-Rad, 1703940). After the transfer, PVDF membrane was incubated with a blocking solution (5% milk in TBST) for 30 min. After blocking, the membrane was incubated overnight at 4°C with the indicated primary antibody. Antibodies used in this study are listed in Table S2. Western blots were visualized using a ChemiDoc^TM^ Touch (Bio-Rad), an Azure 600 (Azure Biosystems), or an iBright CL1500 (Invitrogen) imaging system. Densitometry for western blots was performed using ImageJ (https://imagej.nih.gov/ij/).

### Pho8Δ60 assay

The Pho8Δ60 assay was performed as previously described^[47]^ with the following modifications. Colorimetric detection was measured at 405 nm using the SmartReader 96 microplate absorbance reader (Accuris, MR9600). The values were normalized to the protein content of each sample determined by the Pierce BCA Protein Assay kit (Thermo Scientific, PI23227) as described^[50]^.

### RNA extraction and real-time quantitative PCR (RT-qPCR)

RNA extraction and RT-qPCR were performed as previously described^[38,50]^. Yeast cells were cultured in YPD to mid-log phase and then shifted to SD-N (1 h) to induce autophagy. Cells (1 OD_600_ unit) were collected, and the pellets were immediately flash frozen in liquid nitrogen. Total RNA was extracted using an RNA extraction kit (NucleoSpin RNA; Clontech, 740955.50). Reverse transcription was carried out using the High-Capacity cDNA Reverse Transcription Kit (Applied Biosystems/Thermo Fisher Scientific, 4368814). For each sample, 1 µg RNA was used for cDNA synthesis. RT-qPCR was performed using the Power SYBR Green PCR Master Mix (Applied Biosystems/Thermo Fisher Scientific, 4367659) in a CFX Opus 96 (Bio-Rad, 12011319) real-time PCR machine. For all RT-qPCR experiments, melt curves were run after the PCR cycles to verify primer specificity. Relative gene expression was calculated using the 2^−ΔΔCT^ method^[86]^ and normalized as indicated. Primer sequences are included in Table S3.

### Statistical analysis

The two-tailed unpaired Student’s *t* test was used to determine statistical significance with GraphPad Prism (GraphPad Software, USA) unless otherwise indicated. For all figures, *p* values are as follows: *p < 0.05; **p < 0.01; ***p < 0.001; **** *p* < 0.0001. A *p* value of <0.05 was considered significant.

## Supplementary Material

Supplemental Information.pdf

## Data Availability

All data that contributed to this manuscript are available from the corresponding author upon reasonable request.

## Abbreviations

GFP: green fluorescent protein
NMD: nonsense-mediated mRNA decay
NLS: nuclear localization signal
PA: protein A
PAS: phagophore assembly site
PCR: polymerase chain reaction
PE: phosphatidylethanolamine
PTC: premature termination codon
RT-qPCR: real-time quantitative PCR
SD: standard deviation
TOR: target of rapamycin
*ts*: temperature-sensitive
uORF: upstream open reading frame
UTR: untranslated region
WT: wild-type
