# Supplementary material for: The nonsense-mediated mRNA decay factor Upf3 negatively regulates bulk autophagy progression in Saccharomyces cerevisiae
Source: Autophagy Rep. 2026 Feb 6;5(1):2623730. doi: 10.1080/27694127.2026.2623730 (PMC12885402; doi:10.1080/27694127.2026.2623730)
Supplement: Supplemental Information.pdf [file KAUO_A_2623730_SM1996.pdf]

Supplemental Information

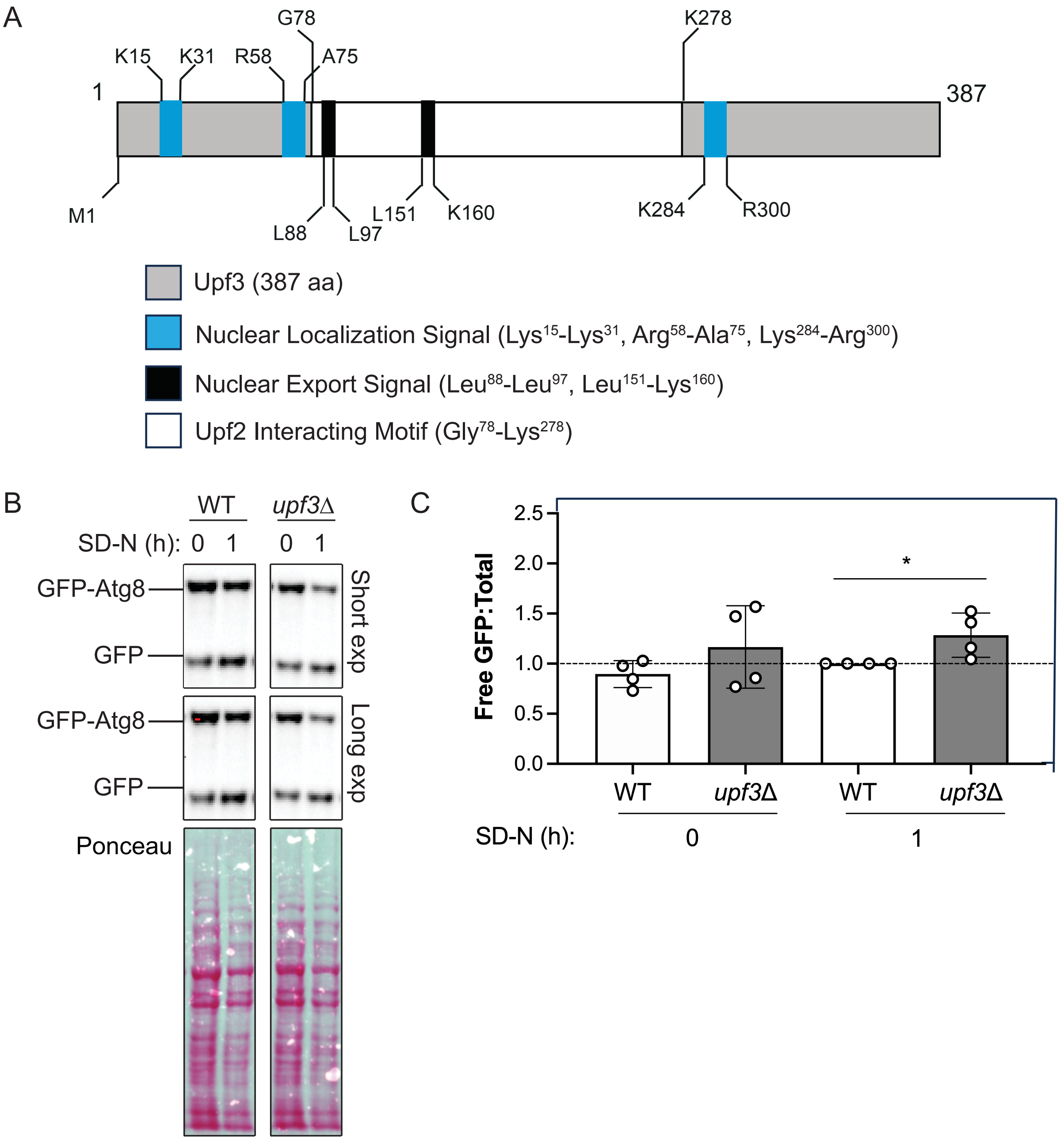

Figure S1. Loss of *UPF3* enhances nonselective autophagy. (**A**) Schematic of the full-length Upf3 protein, including 387 amino acid residues. Nuclear localization signals (NLS; Lys15-Lys31, Arg58-Ala75, and Lys284-Arg300), nuclear export signals (NES; Leu88-Leu97 and Leu151-Lys160) and the Upf2-interaction motif (Gly78 to Lys278) are shown. (**B**) WT (BY4742) and *upf3*Δ cells transformed with a centromeric plasmid expressing GFP-Atg8 under its own promoter were grown to mid-log phase in SD-Ura selective media (Takara) and then starved for nitrogen (SD-N) for 0 and 1 h. Protein extracts were analyzed by SDS-PAGE, stained for total protein (Ponceau S), and then blotted with anti-GFP antibody. Two exposures are included for GFP ("short" and "long"). An irrelevant lane was removed from the blot image. A representative blot is shown (n=4). (**C**) Densitometry of blots represented in (**B**). Processed GFP-Atg8 was calculated by determining the ratio of GFP:total GFP-Atg8 (sum of free GFP and full-length GFP-Atg8). Results shown are relative to the WT strain during starvation (1 h SD-N), which was set to 1. Error bars represent standard deviation (SD; *$p<0.05$). Also see Tables S1 and S2.

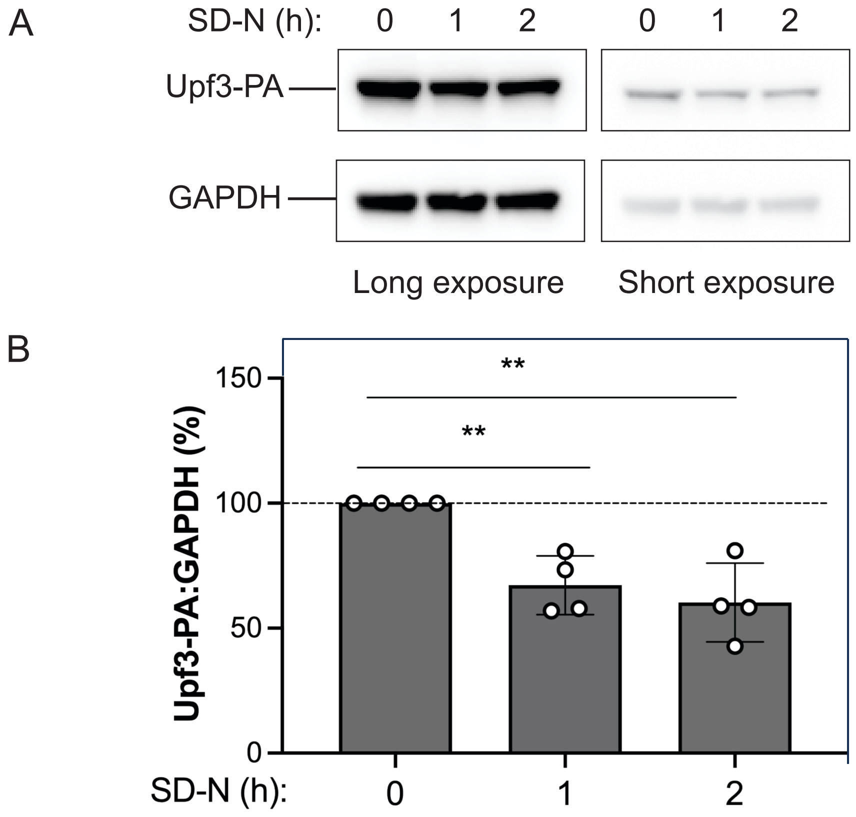

Figure S2. Upf3-PA fusion protein expression levels decrease after autophagy induction. (**A**) Cells endogenously expressing Upf3-PA (EDA310) were grown in YPD to mid-log phase and then starved (SD-N) for 0, 1, or 2 h. Protein extracts were analyzed by SDS-PAGE and blotted with anti-PA or anti-GAPDH (loading control) antibodies. Two exposures are included for Upf3-PA and GAPDH ("long" and short"). (**B**) Densitometry of blots represented in (**A**). The percentage of Upf3-PA:GAPDH was quantified (n=4). Error bars represent SD (**$p<0.01$). Also see Tables S1 and S2.

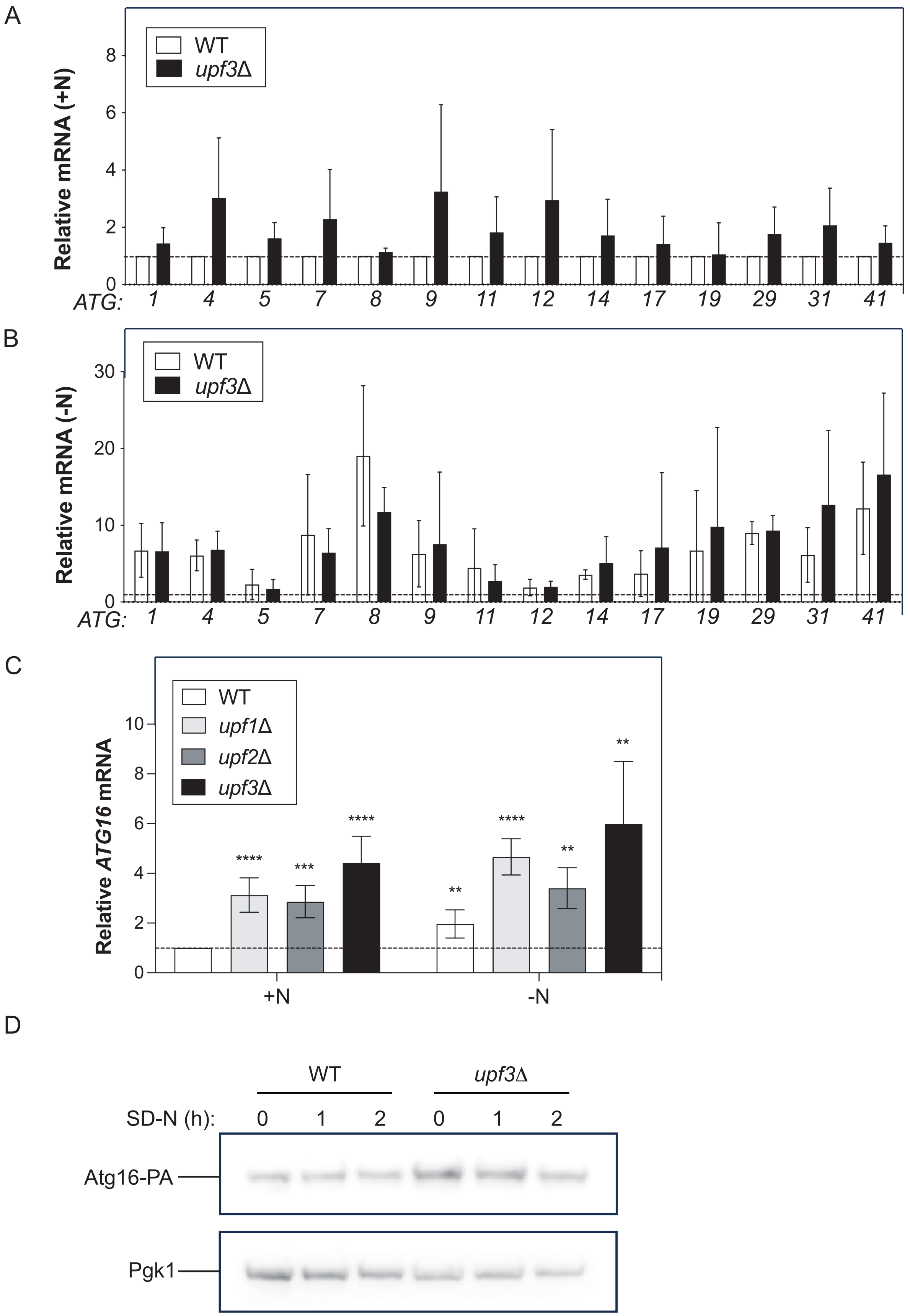
A
WT
upf3Δ
Relative mRNA (+N)
0
2
4
6
8
ATG:
1
4
5
7
8
9
11
12
14
17
19
29
31
41
B
WT
upf3Δ
Relative mRNA (-N)
0
10
20
30
ATG:
1
4
5
7
8
9
11
12
14
17
19
29
31
41
C
WT
upf1Δ
upf2Δ
upf3Δ
Relative ATG16 mRNA
0
2
4
6
8
10
****
***
****
**
****
**
**
+N
-N
D
WT
upf3Δ
SD-N (h):
0
1
2
0
1
2
Atg16-PA
Pgk1

Figure S3. NMD factors negatively regulate *ATG16* expression. (**A**) WT (WLY176) and *upf3*Δ (EDA164) cells were grown to mid-log phase in YPD (+N). Total RNA was extracted, and RT-qPCR was performed. Results are shown relative to the level of mRNA expression in WT cells under rich conditions (+N), which was set to 1. The geometric mean of *TFC1* and *SLD3* were used to quantify relative expression levels. (**B**) WT (WLY176) and *upf3*Δ (EDA164) cells were starved for nitrogen (-N) for 1 h. Total RNA was extracted and analyzed as in (**A**). Results shown in (**A**) and (**B**) are the mean of 4 independent experiments. (**C**) WT (WLY176), *upf1*Δ (EDA138), *upf2*Δ (EDA165), and *upf3*Δ (EDA164) cells were grown to mid-log phase in YPD (+N), then starved for nitrogen (-N) for 1 h. Total RNA was extracted, and RT-qPCR was performed. Results are shown relative to the level of *ATG16* mRNA expression in WT cells under rich conditions (+N), which was set to 1. The geometric mean of *TFC1* and *SLD3* were used to quantify relative expression levels. Results shown are the mean of at least 5 independent experiments. For (**A**)–(**C**), error bars represent standard deviation (SD; $^{**}p<0.01$; $^{***}p<0.001$; $^{****}p<0.0001$). (**D**) Short exposure for blot image shown in Figure 4B. WT (EDA325) and *upf3*Δ (EDA327) cells endogenously expressing Atg16-PA were grown to mid-log phase in YPD and then nitrogen-starved (SD-N) for 0, 1, or 2 h. Protein extracts were analyzed by SDS-PAGE and blotted with anti-PA or anti-Pgk1 (loading control) antibodies (n=4). Also see Tables S1, S2, and S3.

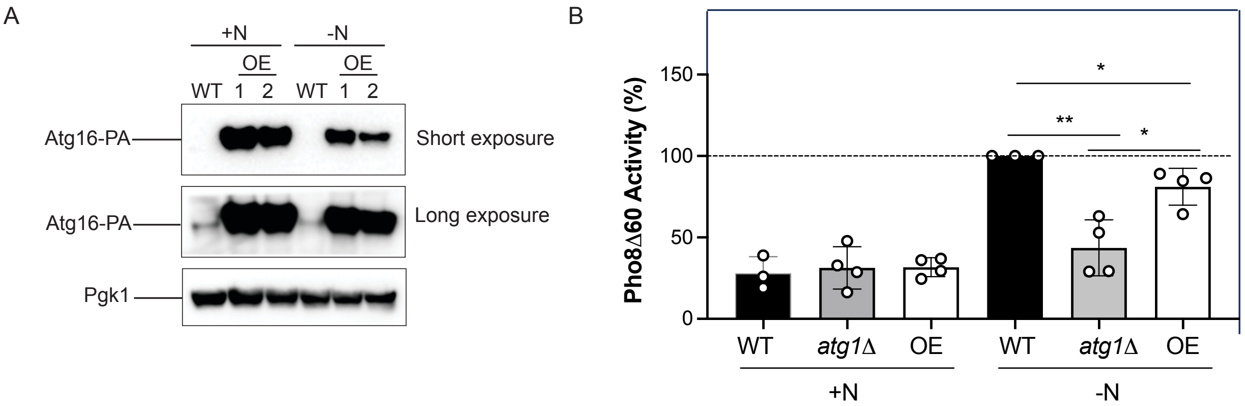

Figure S4. The effect of Atg16 overexpression on Pho8Δ60 activity. (**A**) WT cells endogenously expressing Atg16-PA (EDA325) and two independent preparations (1 and 2, respectively) of cells overexpressing Atg16-PA (*ZEO1p-ATG16-PA*; SBN19) were grown to mid-log phase in YPD (+N) and then starved for nitrogen (–N) for 2.5 h. Protein extracts were analyzed by SDS-PAGE and blotted with anti-PA or anti-Pgk1 (loading control) antibodies (n=4). Two exposures are included for Atg16-PA (“short” and “long”). (**B**) WT (EDA325), *atg1*Δ cells (WLY192), and cells overexpressing Atg16 (OE Atg16; SBN19) cells were grown to mid-log phase in YPD (+N) and then starved for nitrogen (–N) for 2.5 h. The Pho8Δ60 activity was measured and normalized to the activity of starved WT cells at 2.5 h, which was set at 100%. Error bars represent SD (*$p<0.05$; **$p<0.01$). Also see Tables S1 and S2.

Table S1. Strains used in this study.

Name	Genotype	Reference
BY4742	MATα *his3Δ1 leu2Δ0 lys2Δ0 ura3Δ0*	Horizon Discovery
EDA138	WLY176, *upf1Δ::TRP1*	This study
EDA164	WLY176, *upf3Δ::URA3*	This study
EDA165	WLY176, *upf2Δ::KANMX*	This study
EDA283	WLY176, *upf1Δ::TRP1 upf2Δ::URA3 upf3Δ::KANMX*	This study
EDA284	WLY176, *ATG8p-GFP-ATG8(405)::LEU2*	This study
EDA285	EDA283, *ATG8p-GFP-ATG8(405)::LEU2*	This study
EDA302	JMY347, *upf3Δ::KANMX*	This study
EDA310	SEY6210, *UPF3-PA::KANMX*	This study
EDA325	WLY176, *ATG16-PA::KANMX*	This study
EDA327	EDA164, *ATG16-PA::HIS3*	This study
EDA328	JMY347, *atg13Δ::HIS5*	This study
EWA013	SEY6210, *UPF3-3xHA::HIS3*	This study
JMY347	SEY6210, *pho13Δ ZEO1p-pho8Δ60, CUP1p-GFP-ATG8(405)::LEU2*	[1]
SBN19	EDA325, *ZEO1p-ATG16::HIS5*	This study
SEY6210	MAT*α his3Δ200 leu2-3,112 lys2-801 suc2-Δ9 trp1Δ901 ura3-52*	[2]
upf3Δ	BY4742, *upf3Δ::KANMX*	Horizon Discovery
WLY176	SEY6210, *pho13Δ pho8::pho8Δ60*	[3]
WLY192	WLY176, *atg1Δ::HIS5*	[3]
YAB369	YTS158, *atg8Δ::HIS5*	[4]
YTS158	BY4742, *pho13Δ::KANMX pho8::pho8Δ60*	[5]

Table S2. Antibodies used in this study.

Name	Catalog number	Concentration	Source
mouse monoclonal anti-Atg8 (G-10)	sc-373963	1:1,000	Santa Cruz Biotechnology
mouse monoclonal anti-GAPDH (1E6D9)	60004-1-Ig	1:20,000	Proteintech
mouse monoclonal anti-GFP (JL-8)	632381	1:3,000	Clontech
rabbit polyclonal anti-HA tag	51064-2-AP	1:10,000	Proteintech
rabbit polyclonal anti-peroxidase (anti-PA) antibody	323-005-024	1:30,000	Jackson Immunoresearch
mouse monoclonal anti-Pgk1 (22C5D8)	459250	1:5,000	Invitrogen

Table S3. RT-qPCR primers used in this study.

Primer	Sequence (5’ to 3’)	Reference
ATG1-F	ATCTAAGATGGCCGCACATATG	[6]
ATG1-R	AGGGTAGTCACCATAGGCATTC	[6]
ATG4-F	GGTGCAATATTCCCACACACAAC	[7]
ATG4-R	CTCTACAAGGACGCCTTCTAC	[7]
ATG5-F	TCGGTCAACGAAGCTCGAAA	[7]
ATG5-R	GATGAGCGGTATATGTCGCG	[7]
ATG7-F	ATGAGCATTGTCCAGCATGTAG	[6]
ATG7-R	GACCTCCTGCTTTATGACTGAC	[6]
ATG8-F	GAAGGCCATCTTCATTTTTGTC	[6]
ATG8-R	TTCTCCTGAGTAAGTGACATAC	[6]
ATG9-F	CGTACTAACAGAGTCTTTCCTTG	[6]
ATG9-R	CTAAGACACCACCCTTATTGAG	[6]
ATG11-F	CTTTGGAACATTTCGTAGCAGATAGCCC	[7]
ATG11-R	CTCCCTGGTATGAAACCACAACCGC	[7]
ATG12-F	TGGCACTTATGAACAGGAAGAG	[7]
ATG12-R	AACTGCCCAATAGAACCAATG	[7]
ATG14-F	TACTGGACCAGTACGATGTG	[6]
ATG14-R	TGCAGGATGTCCTCTTTGTG	[6]
ATG16-F	GGTTGAATGACGAATTGATTAGT	[7]
ATG16-R	CGCTGTTCATGGCTTCTGTC	[7]
ATG17-F	GCTCAGGACCAGGAAGAACG	[7]
ATG17-R	TCGTCAATTTTACCGGGCCA	[7]
ATG19-F	TTAGAGGCCGGCTCCACTAT	[7]
ATG19-R	GGCTCTTGGGATGCCTGTAA	[7]
ATG29-F	ATGAGGCGTTACAACATTTGC	[6]
ATG29-R	TCGTCATCTGAACTACCGCAC	[6]
ATG31-F	TCAAAGAGCACGGACGGAAG	[7]
ATG31-R	GCTGTCAAAGCAGCATCACT	[7]
ATG41-F	TGGAATTTAACGGCAACGCC	This study
ATG41-R	TTCTGTTGTGCGACATTGGC	This study
1-UPF3-F	TGAGGAAGCGGGTAAAGAGG	This study
1-UPF3-R	TGACTGAGGCTGAGAGGAGT	This study
2-UPF3-F	ACTCCTCTCAGCCTCAGTCA	This study
2-UPF3-R	TACCCGTGGTACGAAAAGGT	This study
SLD3-F	CGCAACTTCAAAGCATCATTGAATCGC	[8]
SLD3-R	GGGGCTTATTAGTGGGAGTAGAGG	[8]
TFC1-F	GCTGGCACTCATATCTTATCGTTTCACAATGG	[9]
TFC1-R	GAACCTGCTGTCAATACCGCCTGGAG	[9]

Supplemental References

[1] Wen X, Gatica D, Yin Z, et al. The transcription factor Spt4-Spt5 complex regulates the expression of ATG8 and ATG41. Autophagy. 2019 Sep 8:1-14.

[2] Robinson JS, Klionsky DJ, Banta LM, et al. Protein sorting in Saccharomyces cerevisiae: isolation of mutants defective in the delivery and processing of multiple vacuolar hydrolases. Molecular and cellular biology. 1988 Nov;8(11):4936-48.

[3] Kanki T, Wang K, Baba M, et al. A genomic screen for yeast mutants defective in selective mitochondria autophagy. Mol Biol Cell. 2009 Nov;20(22):4730-8.

[4] Delorme-Axford E, Abernathy E, Lennemann NJ, et al. The exoribonuclease Xrn1 is a post-transcriptional negative regulator of autophagy. Autophagy. 2018;14(5):898-912.

[5] He C, Song H, Yorimitsu T, et al. Recruitment of Atg9 to the preautophagosomal structure by Atg11 is essential for selective autophagy in budding yeast. J Cell Biol. 2006 Dec 18;175(6):925-35.

[6] Bernard A, Jin M, Gonzalez-Rodriguez P, et al. Rph1/KDM4 mediates nutrient-limitation signaling that leads to the transcriptional induction of autophagy. Curr Biol. 2015 Mar 2;25(5):546-55.

[7] Hu G, McQuiston T, Bernard A, et al. A conserved mechanism of TOR-dependent RCK-mediated mRNA degradation regulates autophagy. Nature cell biology. 2015 Jul;17(7):930-42.

[8] Bernard A, Jin M, Xu Z, et al. A large-scale analysis of autophagy-related gene expression identifies new regulators of autophagy. Autophagy. 2015 Nov 02;11(11):2114-2122.

[9] Teste MA, Duquenne M, Francois JM, et al. Validation of reference genes for quantitative expression analysis by real-time RT-PCR in Saccharomyces cerevisiae. BMC molecular biology. 2009 Oct 30;10:99.
